# Multi-Criteria Decision Analysis Tools for Prioritising Emerging or Re-Emerging Infectious Diseases Associated with Climate Change in Canada

**DOI:** 10.1371/journal.pone.0068338

**Published:** 2013-08-07

**Authors:** Ruth Cox, Javier Sanchez, Crawford W. Revie

**Affiliations:** 1 Atlantic Veterinary College, University of Prince Edward Island, Charlottetown, Prince Edward Island, Canada; University of Ottawa, Canada

## Abstract

Global climate change is known to result in the emergence or re-emergence of some infectious diseases. Reliable methods to identify the infectious diseases of humans and animals and that are most likely to be influenced by climate are therefore required. Since different priorities will affect the decision to address a particular pathogen threat, decision makers need a standardised method of prioritisation. Ranking methods and Multi-Criteria Decision approaches provide such a standardised method and were employed here to design two different pathogen prioritisation tools. The opinion of 64 experts was elicited to assess the importance of 40 criteria that could be used to prioritise emerging infectious diseases of humans and animals in Canada. A weight was calculated for each criterion according to the expert opinion. Attributes were defined for each criterion as a transparent and repeatable method of measurement. Two different Multi-Criteria Decision Analysis tools were tested, both of which used an additive aggregation approach. These were an Excel spreadsheet tool and a tool developed in software ‘M-MACBETH’. The tools were trialed on nine ‘test’ pathogens. Two different methods of criteria weighting were compared, one using fixed weighting values, the other using probability distributions to account for uncertainty and variation in expert opinion. The ranking of the nine pathogens varied according to the weighting method that was used. In both tools, using both weighting methods, the diseases that tended to rank the highest were West Nile virus, Giardiasis and Chagas, while Coccidioidomycosis tended to rank the lowest. Both tools are a simple and user friendly approach to prioritising pathogens according to climate change by including explicit scoring of 40 criteria and incorporating weighting methods based on expert opinion. They provide a dynamic interactive method that can help to identify pathogens for which a full risk assessment should be pursued.

## Introduction

Global climate change is impacting the incidence and distribution of infectious diseases [1]. Canada and the arctic regions are likely to experience greater rates of change than many other regions of the world due to northern latitude and large landmass [2], [3]. There will likely be warmer temperatures, more rainfall, more frequent droughts, and extreme weather events such as hurricanes and tornadoes [4], [5]. These changes in climate are predicted to cause some pathogens to appear in a population (‘emerge’) or cause an existing pathogen to rapidly increase in incidence or geographic range (‘re-emerge’) [6].

Decision makers need to identify the diseases that are most likely to emerge or re-emerge (referred to as ‘emerging’ in the remainder of this paper) in response to climate change as an aid to focusing disease prevention and control measures. This kind of decision making process needs to consider a large number of characteristics (or criteria) of a pathogen or a disease that account for the features of the epidemiological triad, namely the agent, the host and the environment. Objective and transparent methods are therefore required to address this multi-dimensional problem, so that intelligence from a number of sources and the influence of stakeholders with different agendas can be synthesised and so that future actions can be justified.

Considerable research has been carried out to identify the key characteristics of potential emerging infectious diseases and attempts have been made to prioritise these pathogens in terms of their risk of emergence or impact in some countries [7], [8]. In Canada current methods of prioritising potential disease risks are often based on subjective ‘horizon scanning activities’, a non-systematic approach of evaluation, based on personal opinion. However, recent work by the authors identified a number of criteria that can be used to prioritise emerging pathogens in the Canadian context and highlighted that a more standardised approach is required [9].

The objective of our work is to design and test a standardised method to prioritise infectious diseases of humans and animals that may emerge in Canada in response to climate change. To do this we consider both the likelihood of emergence and the impact of a disease if the pathogen were to emerge. There are a number of methods that can be used to standardise decision making, one such method that we used here is Multi-Criteria Decision Analysis (MCDA). MCDA was chosen because it provides a systematic way to integrate information from a range of sources and a structured method of comparing and ranking alternative decisions [10]. There are a range of MCDA approaches (for review see [10]–[12]), and here we tested two alternatives.

The first was a simple approach using an additive aggregation model [12], and used a spreadsheet as a platform. This approach has proven useful for priority setting in health policy in the Netherlands and for assessing food safety and disease risks in salmon farming in the UK [13], [14]. It was chosen because it allowed us to incorporate a transparent and easily altered scoring system and to integrate uncertainty in the method of criteria weighting. The second tool employed an MCDA approach called ‘MACBETH’ (‘Measuring Attractiveness by a Categorical Based Evaluation Technique’), which also uses an additive aggregation approach. MACBETH was chosen for its ability to establish quantitative measurement scales based on qualitative judgements. That is, it only required the user to describe the difference between the two pathogens qualitatively (pathogen x is more important than pathogen y for this criteria). While the MACBETH approach has been used in decision analysis in other subject areas, for example in career choice [15], the method has only very recently been applied to disease prioritisation [16], [17]. We chose two different approaches because the features described above were not available in a single platform. Both tools aim to standardise the multi-dimensional, and in some cases, subjective nature of decision making in an explicit and transparent way. Our purpose was not to explicitly compare the two methods, but to use them to assess whether MCDA is a useful approach to this type of problem.

## Methods

### Ethics statement

The study protocol, including the written consent of all participants, was approved by the University of Prince Edward Island Research Ethics Board (REB Reference #6003938).

Development of both decision tools involved the following steps:

- Identification of criteria that can be used to prioritise pathogens.
- Assignment of attributes to each criterion.
- Expert elicitation to evaluate criteria and criteria attributes
- MCDA tool design.
- Criteria weighting.
- Assignment of values to criteria attributes.
- Calculation of total score for a pathogen.

### Identification of criteria that can be used to prioritise pathogens

We identified 40 criteria that might be used to prioritise potential emerging pathogens in Canada. Criteria were identified from published literature, discussion with experts from universities and government agencies, and where possible were informed by previous disease prioritisation studies [9].

For simplicity, criteria were divided into five groups:

Group A: Disease epidemiology (12 criteria, named A1 to A12).

Groups B: Ability to monitor, treat and control disease (5 criteria, B1 to B5).

Group C: Influence of climate change in Canada (12 criteria, C1 to C12).

Group D: Burden of disease (8 criteria, D1 to D8).

Group E: Economic and social impact (3 criteria, E1 to E3).

The criteria in groups A, B and C measure the likelihood of pathogen emergence in Canada, while groups D and E measure pathogen impact. Our study focuses on the likelihood of pathogen emergence in response to climate change, however criteria related to pathogen impact were also included since they are a necessary part of prioritisation [9].

### Assignment of attributes to each criterion

**Figure 1:**
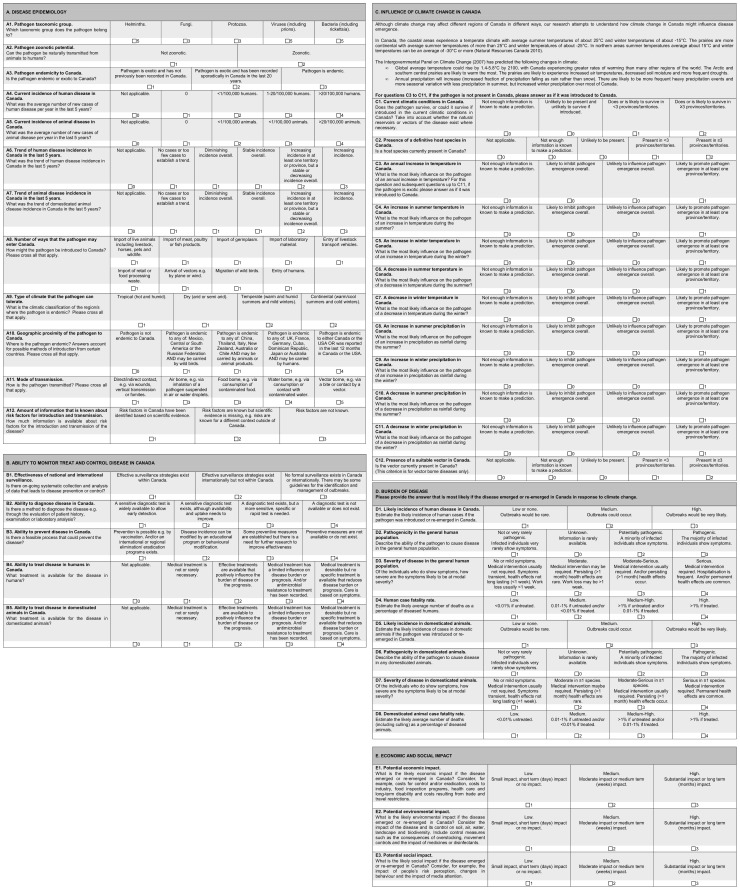
Questionnaire designed to collect expert opinion about infectious disease characteristics for disease prioritisation in Canada. Left hand column: criteria; right hand columns: criteria attributes. Numbers next to tick boxes indicate the value assigned to each attribute in the spreadsheet tool.

In order to standardise the pathogen prioritisation process attributes were assigned to each criterion (Figure 1). The attributes were based on published literature and aimed to be as quantitative as possible. Attributes for some criteria are self-explanatory; we therefore only provide explanation where detail about source information is necessary. Numbers in brackets, e.g. (A1), refer to the criteria that are being described.

#### Group A: Disease epidemiology

The current incidence of human and animal disease (A4 and A5) and the trend of human and animal disease incidence (A6 and A7) focused on a 5 year time scale to assess how recent changes might be relevant in disease emergence. Attributes for (A4) and (A5) were described per 100,000 humans or animals in line with disease incidence reporting by the Public Health Agency of Canada (PHAC) [18]. The aim of criteria A8 was to establish the number of possible entry routes of a pathogen to Canada, following [19], [20]. Attributes for criterion (A9) were derived from the Köppen Climate Classification System, which categorizes regions into four main types (tropical, dry, temperate or continental) based on annual and monthly averages of temperature and precipitation [21]. Pathogens that can tolerate a temperate or continental climate (the climate of the majority of inhabited Canada) were considered a higher risk for emergence than pathogens that are endemic to dry or tropical regions. It is possible to select more than one attribute for this criterion, because pathogens that can tolerate a range of climates are more of a risk than those that can only tolerate one type of climate.

The aim of criterion (A10) was to differentiate between pathogens that are geographically close to Canada and those that are more distant. The attributes considered both pathogen endemicity and the potential method of introduction to Canada. The highest risk pathogens were those with the closest proximity i.e. those that are endemic to Canada or the USA or that were reported in these countries in the last 12 months. High risk methods of pathogen introduction to countries including Canada have been identified as via human immigration, import of animals or animal products [20] and via wild bird migration [22], [23]. The countries that were the highest risk for introduction of pathogens via animal or animal product imports to Canada (USA, China, Thailand, Italy and New Zealand) were those that exported the most animal and animal products into Canada between 2004 and 2009 [24]. Countries that were the greatest risk for human introduction of a pathogen (Mexico, UK, France, Cuba and Dominican Republic, Germany, Japan and Australia) were those where most visitors to Canada arrived from or were the most visited overseas countries in 2009 [25]. The regions where bird migration was considered the highest risk were Mexico, Central or South America and the Russian Federation, since bird migration is generally a north and south movement [26]. The modes of transmission described in (A11) were identified based on [1] and following discussion with experts at PHAC.

#### Group B: Ability to monitor, treat and control disease

The attributes for (B1) to (B5) were designed following other prioritisation work [7], [8], [27] and discussion with medical and veterinary researchers. The minimum and maximum attributes represent the best and worst case scenarios, intermediate attributes describe scenarios in which current procedures could be improved. ‘Not applicable’ attributes were provided for criteria B4 and B5, because if a disease only occurs in the human population, then treatment in the animal population is not relevant (and vice versa) and the ‘not applicable’ attribute can be selected.

#### Group C: Influence of climate change

In Canada specifically, predictions of temperature and precipitation changes are well documented, and experts were provided with an estimate of the magnitude of changes (introductory segment of Section C, Figure 1). The attributes for criteria (C3) to (C11) quantified pathogen emergence in simple terms and accounted for both temporal and/or spatial changes. Although climate change may affect different regions of Canada in different ways, information about the variation in climate in different geographical regions of Canada was captured simplistically by assessing whether a pathogen might emerge in at least one province or territory.

#### Group D: Burden of disease

Section D included criteria about disease incidence (D1 and D5), pathogenicity (D2 and D6), severity (D3 and D7) and fatality (D4 and D8) in the human and domesticated animal populations respectively. The domesticated animal population was specified since estimation of burden in wildlife populations was beyond the scope of this research. These criteria referred to the likely impact if a pathogen were to emerge in Canada. Attributes for criteria were defined by incorporating aspects of other prioritisation exercises [7], [8], [27], [28]. The case fatality rate attributes (D4 and D8) were defined both descriptively (low, medium, high) and quantitatively, e.g. a medium fatality rate corresponds to a fatality of 0.01 to 1% if untreated, and/or <0.01% if treated. Percentages were chosen after consulting annual average fatality rates of a number of diseases, and reviewing other assessments [29], [30].

#### Group E: Economic, environmental and economic impact

The attributes for economic, social and environmental impact were simple (3-tiered), in order to gain a sense of the potential impact without including detailed definitions. Definitions were based on previous prioritisation exercises [7], [20], [31]. Economic impact included costs for control, costs to industry and costs relating to healthcare. Environmental impact related to the impact of the disease and the impact of its control. Social impact was included in order to assess how much society cares about the impact of a disease. These definitions were wide ranging and it was therefore most appropriate to measure them on a scale from low to high.

### Expert elicitation to evaluate criteria and criteria attributes

We used expert opinion as an aid to designing the prioritisation tools. There were two phases to the expert elicitation. In phase one experts were asked to participate in criteria selection, in phase two they were asked to evaluate the criteria attributes and definitions. They were asked to comment on criteria and to suggest alterations or additional criteria if necessary. Experts were from academic, government and independent backgrounds and were defined as individuals whose past or present field contains the subject under study i.e. infectious disease epidemiology and/or climate change, following [32], [33]. They were identified through literature and internet searching and via recommendations from other experts as described by the authors [9].

#### Phase one: elicitation of expert opinion about criteria

Phase one has been described in detail in a previous publication [9]. In summary, experts were presented with the list of criteria (Figure 1), however the attributes for the criteria were excluded at that time. For criteria groups A, B and C they were asked ‘is this criterion likely to influence the probability of an infectious disease emerging in Canada?’. Participant were asked to select one answer from: ‘don't know’, ‘not likely’, quite likely, ‘likely’, ‘very likely’ or ‘extremely likely’. For groups D and E they were asked ‘how important is this criterion for prioritising infectious disease in terms of their impact if they did emerge in Canada?’. One answer could be selected from the attributes: ‘don't know’, ‘not important’, ‘quite important’, ‘important’, ‘very important’ or ‘extremely important’. The phrases were ordered on a five-tiered Likert scale according to their meaning and numerical values were not attached. Experts therefore chose a description relative to the other options on the scale.

We wanted to assess the influence of the climate criteria on different pathogen types, because some types are more likely to be influenced by climate than others. We therefore defined four pathogen types based on their mode of transmission and experts were asked to indicate the likely influence of the criteria in group C on each pathogen type: vector-borne, food and water-borne, air-borne and direct/indirect contact pathogens. The results from phase one were used to determine which criteria should be included in a prioritisation tool and also to calculate a weight for each criterion (see ‘criteria weighting’ below).

The experts were also asked to rate their level of expertise about 14 pathogens (Table 1) as either low (limited background knowledge), medium (contributed to some work in this area) or high (e.g. published research or led research projects in this area). These pathogens had been chosen to test the prioritisation tools and were selected as representative examples of types of pathogen or disease (Table 1) according to characteristics such as taxonomic group, zoonotic potential, mode of transmission (direct/indirect contact, air-borne, food and water-borne and vector-borne), endemicity (endemic or exotic), evidence for being influenced by climate [1], and notifiable status in Canada in 2010 [34], [35]; notifiable diseases being those that are of ‘significant importance to human or animal health or to the Canadian economy’ [35].

**Table 1:** Characteristics of pathogens that were selected for prioritisation testing.

Disease*/Pathogen*	Mode of transmission	Taxonomic group	Endemic? (Yes/No)	Zoonotic? (Yes/No)	Notifiable in Canada? (Y/N or R = reportable)	Influenced by climate? (Yes/No)
**Blastomycosis** *	Air borne	Fungus	Y	Y	N	Y
***Blastomyces dermatitidis***						
**Bluetongue**	Vector borne	Virus	N	N	Y or R (type dependent)	Y
***Blue tongue virus***						
**Chagas disease**	Vector borne	Protozoan	N	Y	Y (immediately)	Y
***Trypanosoma cruzi***						
**Chikungunya** *	Vector borne	Virus	N	Y	N	Y
***Chikungunya virus***						
**Cholera**	Water borne	Bacteria	N	Y	Y (immediately)	Y
***Vibrio cholerae***						
**Coccidioidomycosis**	Air borne	Fungus	N	N	Y (annually)	Y
***Coccidiosis imitis***						
**Dengue** *	Vector borne	Virus	N	Y	N	Y
***Dengue fever virus***						
**Foot and Mouth disease**	Direct	Virus	N	Rarely	R	N
***Foot and Mouth disease virus***						
**Giardiasis**	Food/water borne	Protozoan	Y	Y	Y	Y
***Giardia lamblia***						
**Hantavirus Pulmonary Syndrome**	Direct/indirect contact	Virus	Y (Rare)	Y	Y	Y
***Sin Nombre virus***	& Air borne.					
**Lyme disease**	Vector borne	Bacteria	Y	Y	N	Y
***Borrelia spp.***						
**Rift Valley fever** *	Vector borne	Virus	N	Y	R	Y
***Rift Valley fever virus***						
**Streptococcus pneumonia** *	Direct/indirect contact	Bacteria	Y	Y	N	Y
**S** ***treptococcus pneumonia***						
**West Nile virus**	Vector borne	Virus	Y	Y	Y	Y
***West Nile virus***						

Fourteen pathogens were selected of which nine were tested in the prioritisation tools.

* Five were excluded because there were not a sufficient number of individuals to complete a questionnaire. A minimum of three questionnaires were required per pathogen for inclusion.

#### Phase two: Elicitation of expert opinion about criteria attributes

All of the experts who completed criteria weighting (phase one) were then invited to evaluate the criteria attributes. For this, they were sent an electronic questionnaire designed in Microsoft Word 2007 via email. The questionnaire presented the list of 40 criteria plus the criteria attributes (Figure 1) and experts were asked to answer the questionnaire for one pathogen about which they were particularly knowledgeable. This pathogen was selected by the authors according to the participant's judgement of their expertise from phase one. The aim of this phase was to assess whether the criteria attributes were appropriate, rather than to collect information about pathogens, and experts were therefore invited to suggest improvements where necessary.

### MCDA tool design and pathogen prioritisation

The structure of each MCDA tool and the pathogen prioritisation will be described for each tool.

### Excel spreadsheet tool for pathogen prioritisation

#### Spreadsheet tool structure

**Figure 2:**
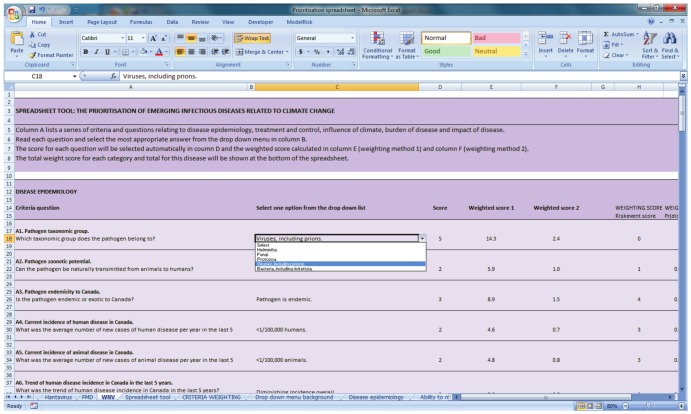
Spreadsheet tool to assess the risk of emergence or re-emergence of infectious diseases associated with climate change.

A spreadsheet tool was developed in Excel (®, Microsoft, Redmond, WA, USA). In summary, the criteria were listed and the criteria attributes were implemented as predefined drop-down selection boxes (Figure 2). Criteria were weighted and attributes were assigned values so that completion of the spreadsheet calculated a score for a pathogen.

#### Criteria weighting

A weight was calculated for each criterion using the expert opinion collected during phase one of the expert elicitation. Two different weighting methods were tested. For weighting method 1, the definitions of likelihood' (‘don't know’, ‘not likely’, ‘quite likely’, ‘likely’, ‘very likely’ or ‘extremely likely’) were assigned values of 0, 0.1, 0.3, 0.5, 0.7 and 0.9. The same values were assigned to the definitions of importance (‘don't know’, ‘not important’, ‘quite important’, ‘important’, ‘very important’, ‘extremely important’). Criteria weight was calculated as the mean value of all experts. The ‘don't know’ responses were included in the calculation because they indicate the amount of uncertainty of the experts. A detailed description of weighting using this method can be found in [9].

The experts weighted the influence of the climate criteria for four different pathogen types (vector-borne, food and water-borne, air-borne and direct or indirect contact pathogens). Four different weights were therefore calculated for each climate criteria – one for each pathogen type.

Weighting method 2 accounted for the variation in expert opinion. Instead of using a single point estimate, weight was modelled as a probability distribution. A single random value for weight was generated from the discrete distribution of likelihood using the Excel add-on ‘*ModelRisk’* (http://www.vosesoftware.com/). Likelihood definitions (ranging from ‘not likely’ to ‘extremely likely’), were converted to a continuous distribution between 0.01 and 1. This was done by converting a weight of ‘not likely’ to a random value of between 0.01 and 0.19, a weight of ‘quite likely’ to a random value between 0.2 and 0.39 etc., in a manner similar to that adopted in [36]. A total of 10,000 iterations were used to capture the weight distribution for each criterion.

#### Assignment of values to criteria attributes

Selecting an attribute for a criterion from the dropdown menu in the spreadsheet generated a predefined quantitative value (Figure 1). For most criteria the attributes could be placed in a naturally ascending order and assigned a value on a linear scale. For example, the four attributes for criterion (A4), (Current incidence of disease in Canada), were: 0, <1/100,000 humans, 1–20/100,000 humans, >20/100,000 humans. By placing these in ascending order, a value of 1, 2, 3 or 4 was assigned to each attribute respectively; a higher risk therefore generating a higher value. Some attributes were assigned a value of 0, when the attribute was ‘not applicable’ or ‘not enough information is known to make a prediction’ or when there was no perceived risk.

There were two criteria where the attributes could not be ranked intuitively. These criteria were (A1): ‘pathogen taxonomic group’ and (A11): ‘mode of transmission’. In these cases, expert opinion was used to rank the attributes and to assign a quantitative value based on the modal ranking as described in [9]. In summary, when experts were asked to rank five taxonomic groups according to how likely they are to be influenced by climate (5 being most likely and 1 being least likely), the modal ranking was: bacteria (5), viruses (5), helminths (5), fungi (3) and protozoa (3). Similarly, the modal ranking of the modes of transmission (A11) was: vector-borne (5), water-borne (4), food-borne (3), air-borne (3) and direct/indirect contact (1).

Attribute values were normalised according to the number of possible attributes i.e. the value was divided by the number of attributes available (known as ‘absolute normalisation’ [37]). This was done so that the relative attractiveness of each attribute was equal following [15].

#### Calculation of total score for a pathogen

The spreadsheet tool was trialed on nine different ‘test’ pathogens. Information about each pathogen was entered into the spreadsheet via the pre-defined drop-down menus. The answers that experts provided during phase two of expert elicitation were used to answer each criterion. If different experts provided different answers for the same pathogen then the modal answer was selected or if two answers were equally common then the highest scoring attribute (worst case scenario) was selected.

The tool calculated the total score for a pathogen as a linear weighted sum of scores. This approach is a simple and common method [11], [12], [38], and is appropriate here because the attribute values were of similar size and scale for each criterion. Thus the score for a pathogen:
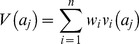
(1)where *V(a_j_)* is the total score for a pathogen *j*, n is the total number of criteria, *w_i_* is the weight assigned to criterion *i, v*_i_(*a_j_*) is the normalised attribute value for criteria *i*, pathogen *j*. We also calculated the linear sum of scores for each group of criteria using the same weighted sum method. Using weighting method 2, the score for each criterion was calculated as the mean of 10,000 iterations.

### MACBETH tool for pathogen prioritisation

#### MACBETH tool structure

**Figure 3:**
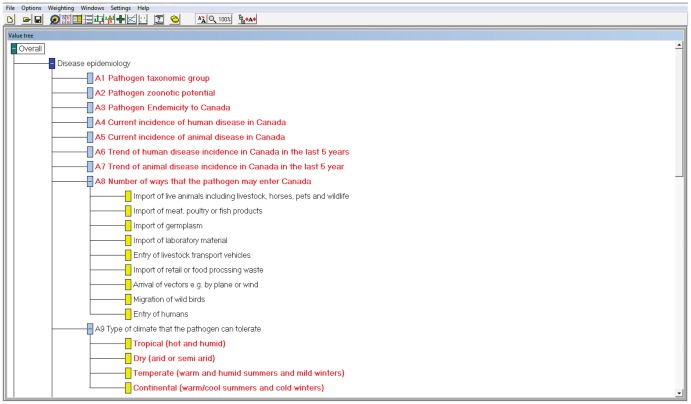
Decision tree structure (showing some of the criteria) developed in the software M-MACBETH. Branches of the decision tree with a light blue branch are criteria, those with a yellow branch are attributes within one criterion.

The MACBETH tool was developed in the software M-MACBETH (version 2.3.0, www.m-macbeth.com, BANA consulting 2010). The criteria were organised into the five criteria groups in a ‘value tree’ (Figure 3).

#### Criteria weighting

Criteria were weighted using weighting method 1 (described for the spreadsheet tool). Weights of all criteria were standardised to sum to 100 by dividing each weight by the sum of all weights and multiplying by 100. Weights ranged from 0.58 to 1.84.

#### Assignment of values to criteria attributes

**Figure 4:**
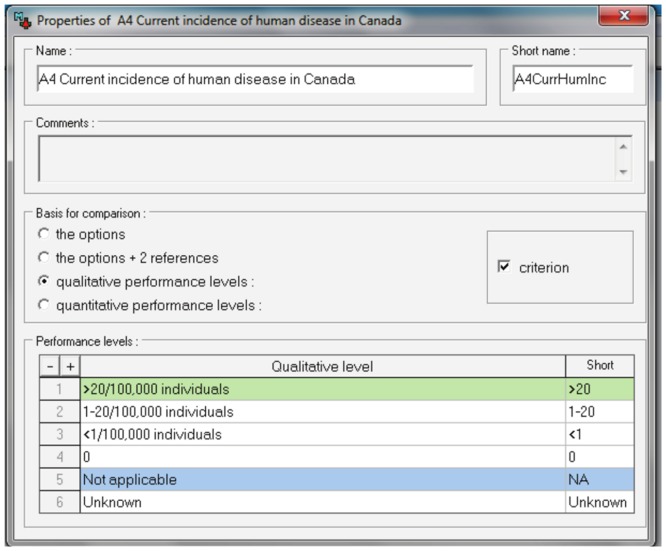
Properties of criteria A4: “Current incidence of human disease in Canada”, showing the 6 different attributes. Lower (blue) and upper (green) act as the scale's arbitrary values of 0 and 100 respectively.

**Figure 5:**
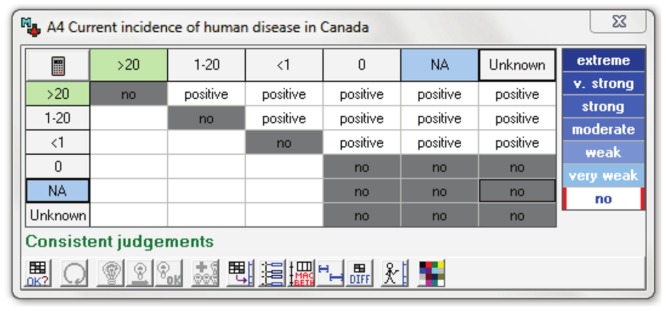
Matrix of attributes for criterion A4 indicating the difference between each attribute. The difference in value of two attributes is either ‘positive’ (i.e. one is greater than the other e.g. >20 is greater than 1–20) or where there is ‘no’ difference in value between two attributes. In this case there is no difference between answers of ‘0’, ‘not applicable’ or ‘unknown’.

A value was assigned to each criterion attribute using an M-MACBETH generated matrix. For example, the attributes for criteria A4, were placed in order of severity (Figure 4) (>20/100,000 humans, 1–20/100,000, <1/100,000, 0, not applicable or unknown) and the difference between each attribute was defined in the matrix (Figure 5). In this case the difference between each attribute was defined as ‘positive’ (meaning that attribute >20/100,000 is more severe than 1–20/100,000, which is more severe than <1/100,000 and so on). The difference between each attribute was of the same magnitude. If two attributes were considered to be equal then the difference was defined as ‘No’ in the matrix.

**Figure 6:**
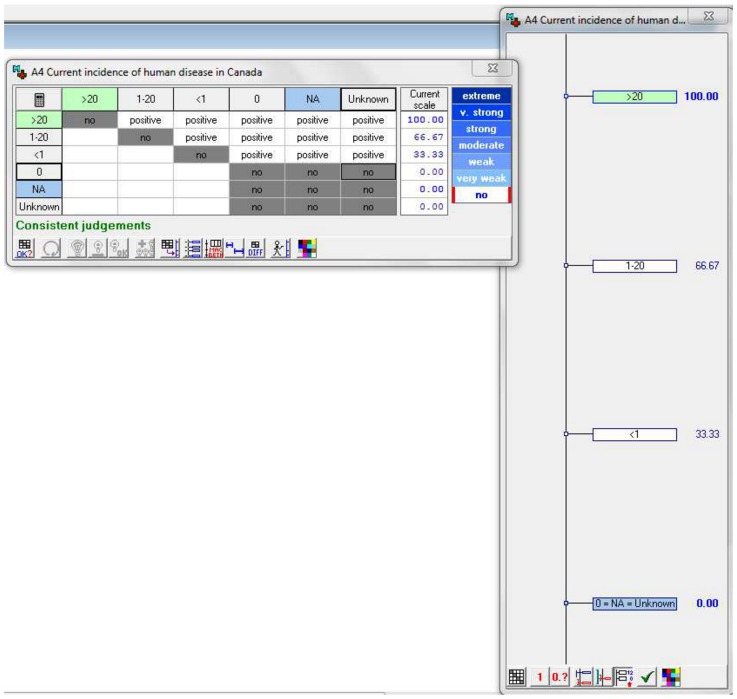
M-MACBETH derived scores as they were allocated to criterion attributes in the matrix.

Once completed, M-MACBETH used this qualitative information to assign a value to each attribute on a scale from 0 to 100 (Figure 6). For criteria A4, therefore, the lowest reference attribute (not applicable) was assigned 0. Since there was no difference between ‘not applicable’, ‘unknown’ and ‘0 incidence’, these attributes were all assigned a value of 0. The highest reference attribute (>20/100,000 individuals) was assigned 100. The attributes between these reference attributes were then assigned values on a linear scale with equal distance between each value. Therefore the second lowest attribute (<1/100,000 individuals) was assigned a value of 33.33 and the next attribute (1–20/100,000 individuals) was assigned a value of 66.67. Values were equally spaced in this case because the differences between each attribute were deemed to be of the same magnitude.

#### Calculation of total score for a pathogen

The MACBETH tool was trialed on nine different ‘test’ pathogens. Information about each pathogen was entered via pre-defined drop-down menus. M-MACBETH calculated the score for each criterion using an additive aggregation model. This was the same fundamental approach as the spreadsheet tool (equation 1), however there were differences in the way that the weight and attribute values were calculated. Firstly in M-MACBETH, the criteria weights were standardised to between 0 and 100. Secondly, M-MACBETH assigned a value of between 0 and 100 to each attribute relative to the other attributes. It did this by assessing the difference between each attribute in the attribute matrix (Figures 5 and 6), (described in ‘assignment of values to criteria attributes’).

### Sensitivity analysis

In order to test the sensitivity of the spreadsheet and MACBETH approaches, pathogen ranking was repeated using ‘reduced’ versions of each tool, which only included the top 10 weighted criteria and excluded all others. The top ten were selected because such a model would represent a relatively quick method to rank pathogens. In addition, to assess the importance of criteria within each group, ‘intermediate’ tools were built by excluding half of the criteria in one group at a time. The criteria that were excluded were those that had the lowest weighting in the group (6 criteria from group A, 3 from group B, 6 from C, 3 from D and 2 from E in turn).

## Results

### Expert response

A total of 64 experts weighted the criteria and detailed discussion of the expert response is presented in [9]. None of the criteria were considered irrelevant to the prioritisation and so none were excluded when the prioritisation tools were built. The five criteria deemed most likely to influence pathogen emergence or impact were ‘potential economic impact’ (E3), ‘severity of disease in the human population’ (D3), ‘human case fatality rate’ (D4), ‘type of climate that the pathogen can tolerate’ (A9) and ‘likely incidence of human disease in Canada’ (D1) [9].

Of the 64 experts who weighted the criteria, 47 completed phase two in which they completed a questionnaire about a specific pathogen (72% response rate). Pathogens were used as ‘test’ pathogens for the prioritisation tools if at least three experts had completed a questionnaire. Nine pathogens were included as ‘test’ pathogens; five were excluded due to lack of expertise. The number of questionnaires completed for the nine ‘test’ pathogen varied between three and eight. During this phase experts suggested some minor alterations to the attributes which were incorporated into the prioritisation tools. These included clarification of wording and the addition of a ‘not applicable’ attribute for some criteria.

### Pathogen prioritisation using the spreadsheet approach

**Figure 7:**
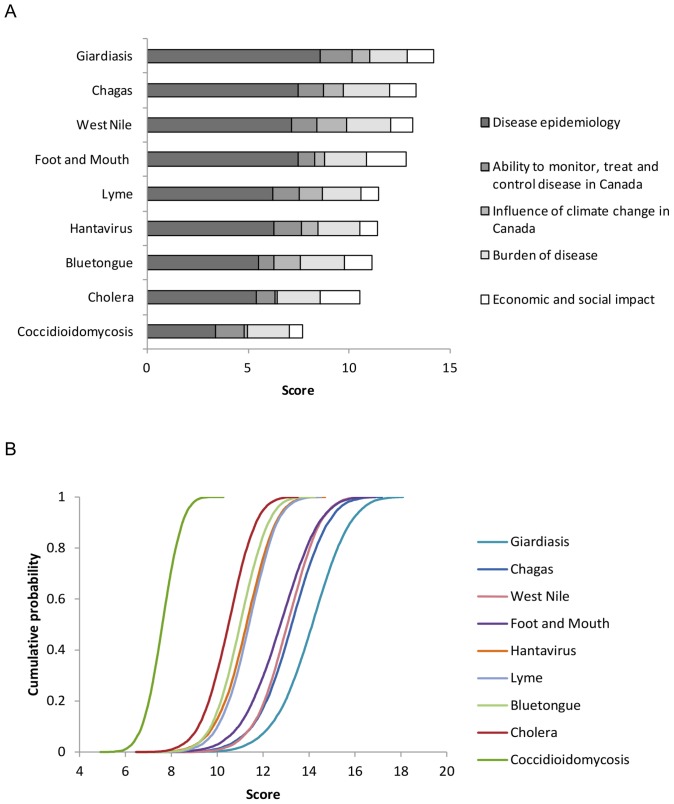
Disease ranking calculated in the spreadsheet tool for nine diseases. A: Criteria were weighted using a fixed mean value based on expert opinion (weighting method 1). The maximum score possible for any disease was 23.7. B: Criteria were weighted using a probability distribution representing the range of expert opinion (weighting method 2). Cumulative probability distribution shows the total score over 10,000 iterations for each disease. The maximum score of a disease was a mean of 23.5 (standard deviation ±2.37, 95^th^ percentile = 27.2 after 10,000 iterations).

Using the spreadsheet tool, the overall ranking of the nine pathogens was the same for both weighting methods. The diseases that ranked the highest overall were Giardiasis, Chagas disease and West Nile virus (Table 2, columns 2 and 4; Figures 7A and B). Bluetongue, Cholera and Coccidioidomycosis ranked the lowest. Within criteria groups, the highest ranking pathogens were similar irrespective of weighting method (Table 3, column 2). Between criteria groups, there was a difference in the high ranking diseases, for example Giardiasis and Chagas disease ranked highly according to disease epidemiology criteria, while West Nile virus and Bluetongue ranked highly according to the influence of climate.

**Table 2:** Ranking of nine diseases according to the two different weighting methods used in the spreadsheet tool and the MACBETH tool.

Rank	Spreadsheet Weighting 1	Reduced spreadsheet Weighting 1	Spreadsheet Weighting 2	Reduced spreadsheet Weighting 2	M-MACBETH Weighting 1	Reduced M-MACBETH Weighting 1
1	Giardiasis	Giardiasis	Giardiasis	Giardiasis	West Nile	West Nile
2	Chagas	Chagas	Chagas	Chagas	Giardiasis	Cholera
3	West Nile	Foot and Mouth	West Nile	Foot and Mouth	Chagas	Chagas
4	Foot and Mouth	West Nile	Foot and Mouth	West Nile	Hantavirus	Lyme
5	Lyme	Lyme	Lyme	Lyme	Lyme	Foot and Mouth
6	Hantavirus	Hantavirus	Hantavirus	Hantavirus	Bluetongue	Giardiasis
7	Bluetongue	Cholera	Bluetongue	Cholera	Foot and Mouth	Hantavirus
8	Cholera	Bluetongue	Cholera	Bluetongue	Cholera	Bluetongue
9	Coccidioidomycosis	Coccidioidomycosis	Coccidioidomycosis	Coccidioidomycosis	Coccidioidomycosis	Coccidioidomycosis

Weighting method 1 used a fixed weight value for each criterion, while weighting method 2 selected a weight from a probability distribution.

The spreadsheet and MACBETH tools contained 40 different criteria. The reduced tools contained the ten most highly weighted criteria.

**Table 3:** Top two ranked diseases per criteria group according to the spreadsheet and MACBETH tools.

Criteria group	Excel Weighting 1 and 2	M-MACBETH Weighting 1
A Disease epidemiology	Giardiasis	Giardiasis
	Chagas	West Nile
B Ability to monitor, treat and control disease	Giardiasis	Giardiasis
	Coccidioidomycosis	Coccidioidomycosis
C Influence of climate	West Nile	West Nile
	Bluetongue	Hantavirus
D Burden of disease	Chagas	Bluetongue
	Bluetongue	Chagas, West Nile^1^
E Economic, social and environmental impact	Foot and Mouth	Cholera
	Cholera	Foot and Mouth

1 Chagas and West Nile were of equal ranking.

**Figure 8:**
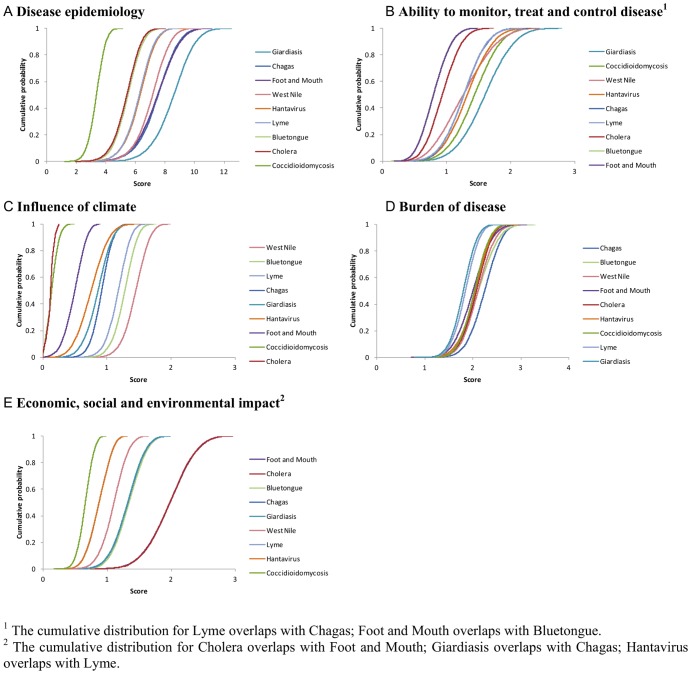
Disease ranking by criteria grouping calculated in the spreadsheet tool for nine diseases. Criteria were weighted using a probability distribution representative of expert opinion. Cumulative probability distribution shows the score for each disease during 10,000 iterations. Legends show pathogen ranking.

Using a probability distribution to weight criteria accounted for variation in expert opinion and also highlighted the expert's uncertainty for some pathogens. For example, there was more uncertainty about Foot and Mouth disease than there was about West Nile virus illustrated by the steeper slope of the cumulative probability for West Nile virus (Figure 7B). This method also highlighted uncertainty within groups of criteria (Figure 8A–E). For example, there was more uncertainty about the influence of climate on Giardiasis than on Chagas disease as illustrated by the steeper cumulative probability of Chagas disease (Figure 8C).

### ‘Reduced’ and ‘Intermediate’ spreadsheet tool results

When the prioritisation was repeated using the ‘reduced’ tool containing ten criteria, there were only small changes in the ranking of pathogens using either weighting method (Table 2, columns 3 and 5 respectively). When the ‘intermediate’ tools were built by excluding half of the criteria with the lowest weightings in one group at a time, there was some change in pathogen ranking, however in all tools the top three pathogens remained in the top three rankings, while the two lowest ranking pathogens remained in the lowest ranks.

### MACBETH pathogen ranking

**Figure 9:**
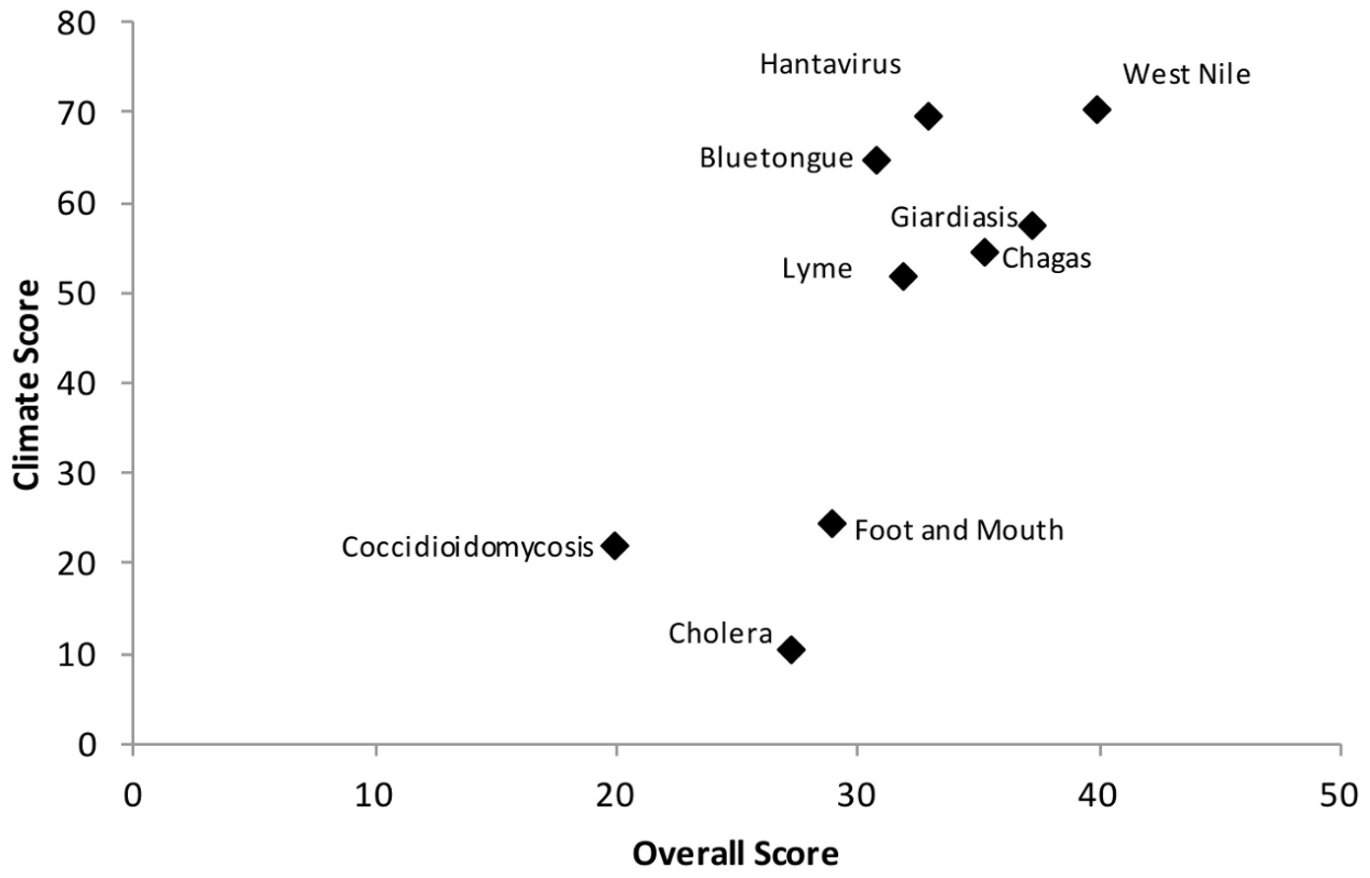
Total score compared to the ‘influence of climate’ score for each of nine diseases in the MACBETH tool. West Nile virus was the highest ranking disease overall and the disease most likely to be influenced by climate.

West Nile virus ranked the highest of all the diseases and Coccidioidomycosis ranked the lowest overall (Table 2, column 6). Different diseases ranked highly within different criteria groups (Table 3, column 3). For example Giardiasis and West Nile virus ranked highly in disease epidemiology. Giardiasis and Coccidioidomycosis were considered the diseases that were the most difficult to monitor, treat and control. Diseases most likely to be influenced by climate were West Nile virus and Hantavirus (Table 3 and Figure 9). Diseases deemed to have the greatest impact on the human and animal population were Chagas disease, West Nile virus and Bluetongue. Cholera and Foot and Mouth disease ranked most highly according to economic, environmental and social impact.

**Figure 10:**
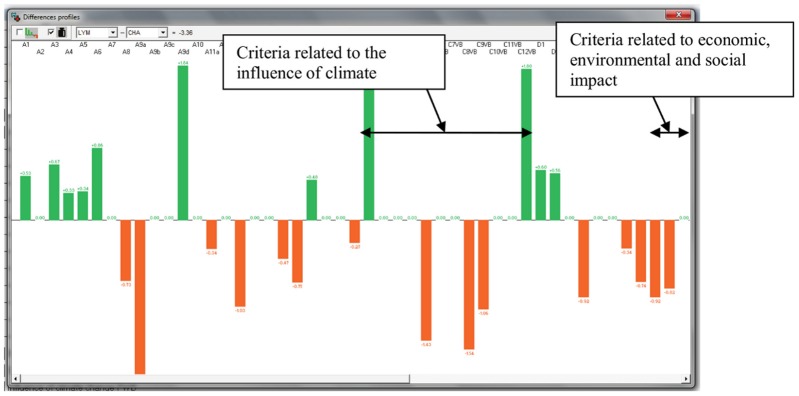
Difference profile of Lyme disease compared to Chagas disease. Bars indicate the difference in the score of two diseases for each criterion. A score of 0 (i.e. no bar) indicates that the two diseases scored the same. A green bar indicates that Lyme scored higher than Chagas, while orange bars indicate that Chagas scored higher than Lyme.

Pair-wise comparison of diseases could be conducted in the MACBETH tool via difference profiles. For example, the difference profile of Lyme disease compared to Chagas disease (Figure 10) highlighted that Lyme disease and Chagas disease tended to be similar in their response to climate (for seven of the twelve climate criteria), while Chagas disease was likely to have a higher economic, social and environmental impact than Lyme disease.

### ‘Reduced’ and ‘Intermediate’ MACBETH tool results

When the prioritisation was repeated using the ‘reduced’ tool that contained the ten most highly weighted criteria, West Nile virus ranked highest and Coccidioidomycosis lowest, as in the complete model (Table 2, column 7). Pathogens that changed rank the most were Cholera which increased in rank to second place, while Giardiasis decreased from second to sixth place.

When the ‘intermediate’ models were built (excluding half of the criteria with the lowest weightings in one group at a time), results were similar to the spreadsheet ‘intermediate’ tools in which there were small changes in pathogen ranking and in all cases the top three pathogens remained in the top three rankings, while the two lowest ranking pathogens remained in the lowest ranks.

### Comparison of the results of the spreadsheet and MACBETH tool

The spreadsheet and the MACBETH tool produced comparable results overall (Table 2). In both tools, the high ranking diseases tended to be Giardiasis, Chagas disease and West Nile virus, while Coccidioidomycosis and Cholera ranked lowest. The only disease that was notably different was Foot and Mouth disease which ranked consistently higher in the spreadsheet tool than in the MACBETH tool. Within criteria groups (Table 3), the only notable difference was in the top ranking diseases within ‘disease epidemiology’. Here Giardiasis ranked highly in both tools, but Chagas disease which ranked second in the spreadsheet tool ranked only sixth in the MACBETH tool.

## Discussion

### Score and ranking

Two different tools were used to rank pathogens based on a total of 40 different criteria. The high ranking diseases (Giardiasis, West Nile virus and Chagas disease) tended to be the same across all tools and weighting methods. Division of criteria into groups proved valuable for determining why pathogens ranked in a particular order. This was especially useful given that our focus was on pathogens that might emerge in response to climate change.

Giardiasis and West Nile virus scored highly for disease epidemiology because they are endemic to Canada (a high risk for re-emergence in these tools), because their current incidence is relatively high in Canada and because they tolerate a variety of climates. Giardiasis and Chagas disease also scored highly because they can be transmitted in a number of ways (direct or indirect contact, food-borne and water-borne; direct or indirect contact, food-borne and vector-borne respectively). The high rank of Chagas disease was also due to the high number of potential routes of introduction to Canada.

Giardiasis and Coccidioidomycosis ranked particularly highly in criteria group B indicating that their surveillance, treatment and control were considered less effective than for most of the other diseases. Conversely the surveillance and diagnosis of the low ranking diseases such as Bluetongue and Foot and Mouth disease were considered relatively effective.

As expected, the diseases that were deemed most influenced by the climate criteria alone were the vector-borne diseases West Nile virus and Bluetongue. These diseases ranked highly due to their modes of transmission and because their emergence was likely to be promoted by increases in summer and winter temperatures and precipitation. The only criteria that were thought to inhibit emergence or where not enough information was available to make a prediction were decreases in summer and winter temperature or precipitation. Not surprisingly our results are in accord with the scientific evidence about the influence of climate on West Nile virus and Bluetongue and whose emergence in the US and Europe respectively has been attributed to the spread of the vector species [39], [40]. Other diseases that also ranked relatively highly for this criteria group included Chagas disease, because the experts thought that emergence would be promoted by increases in temperature and in precipitation, although it is unlikely that the vector is present in Canada. While the relatively high ranking of Chagas was somewhat surprising, literature searches revealed that Chagas disease has recently become of concern for emergence in the US and Canada [41], [42] and that a higher risk is associated with increasing temperatures [41].

Diseases that were deemed least influenced by climate were Foot and Mouth disease, Coccidioidomycosis and Cholera. Foot and Mouth disease was included as a ‘test’ disease because, as a virus that is spread via direct contact, it is unlikely to be influenced by climate and our experts concurred. Coccidioidomycosis, an air-borne fungus, was considered unlikely to be influenced by climate or inhibited by most of the climatic changes. Cholera also generated a low score in this section, although the notable level of uncertainty shown by the experts for at least eight of the criteria undoubtedly contributed to its low rank. This uncertainty reflects the lack of knowledge about the direct influence of climate on Cholera [43]. Although the indirect effects of climate on the emergence of Cholera [44], as well as other diseases [45] have been documented, we did not attempt to capture information about the indirect effects of climate change (e.g. land use, wildlife migration), nor the effects of ‘extreme’ events (e.g. flooding) on disease emergence due to their unpredictability.

Chagas disease, Cholera and West Nile virus ranked the highest for burden of disease because they are pathogenic, cause severe symptoms and high fatality in humans and/or animals. Those with a substantial economic, environmental and social impact if they were to emerge were Foot and Mouth disease and Cholera. In comparison, a disease such as Hantavirus was considered to have a low or medium impact even though it is serious in severity and has a high fatality rate. Its low economic, low environmental and moderate social impact likely reflects that it does not spread between humans (it is spread to humans by rodents) and would therefore affect a small number of individuals in the population compared to a human to human transmissible disease.

There were only small changes in the ranking of diseases between the spreadsheet and MACBETH tools. The most notable difference was the drop in rank of Foot and Mouth disease overall, and the drop in rank of Chagas disease within the ‘disease epidemiology’ group. We attribute these differences to the weighting method, since the weights in M-MACBETH were required to be standardised to sum to 100. This resulted in less distinction between criteria. The other difference was in the method of assigning values to criteria attributes, with M-MACBETH assigning values of 0 and 100. In the case of Foot and Mouth disease, the overall score was mainly due to its disease epidemiology (group A) and its economic, social and environmental impact (group E). Group A contains the greatest number of criteria, and group E contains the most highly weighted criteria. Since weighting in M-MACBETH tended to be more evenly spread than in the spreadsheet tool we attributed the drop in rank to this ‘equalizing’ of weights, which had most influence on group A and group E. In the case of Chagas disease, the drop in rank within group A may also be attributed to the ‘equalizing’ of weights. Within group A, Chagas gained high scores for the highest weighted criteria and low scores for the lowest weighted criteria. A degree of ‘equalizing’ of the weights resulted in the observed drop in rank. Changes to other pathogens were less drastic since other criteria played more of a role in their overall rank.

### Weighting

Criteria weighting is considered an important component of any prioritisation tool and our previous work describes the relative importance of the criteria [9]. While many prioritisation schemes have not incorporated weighting, implying that each criterion has equal importance, Krause showed that disease ranking typically varies between weighted and unweighted criteria [8].

Qualitative weighting (on a scale of ‘likelihood’ or ‘importance’) rather than quantitative weighting was deemed most appropriate because individuals prefer to use imprecise methods such as verbal description of uncertainty when describing events in which the underlying uncertainty is also vague [46]. Since the qualitative descriptions were presented on a scale, there was little opportunity for variation in interpretation by experts in this context. Other studies that have used similar qualitative scales, have generated consistent expert interpretation e.g. a likelihood scale [47], or a low to high scale [19].

Experts were asked to judge the importance of each criterion prior to assessment of criteria attributes. This was done so that we could identify which criteria to include in the prioritisation tools and because this approach has been suggested to increase the objectiveness of the procedure [8]. Although some authors suggest that the entire context of the prioritisation should be presented in one go [48], respondents to the work by Krause supported the separation of the weighting from the actual prioritisation [49]. We believe that our approach simplified the process of criteria selection, because the experts had the opportunity to critique the criteria before moving to phase two in which they critiqued the criteria and attributes together.

There was little variation in the results between the two weighting methods that were tested in the spreadsheet tool. Weighting method 2 provided a more complete description of the subjective nature of the expert opinion and a full analysis of the variability and uncertainty of experts can be found in [9]. The analysis supported the idea that it is not reasonable to expect consensus when tackling difficult-to-predict problems [50] and that finding a method of quantifying uncertainty (as we have done here), rather than removing it from the decision process is an important goal when relying on expert advice [51]. In hindsight it would have been useful to ask experts to indicate their level of certainty in their opinion, and future expert elicitation might benefit by quantifying expert uncertainty, for example by following the ‘Cooke’ method [51], or by using Bayes nets [52].

One drawback of the use of M-MACBETH was the inflexibility of the fixed weighting method and its inability to accommodate a probability distribution. In either tool, the weighting method could be adapted according to the problem being assessed [53]. For example, weights could be modified to account for the interests of the user (e.g. public health practitioner or veterinarian) or to be applicable to a specific region of Canada. This adaptability could prove particularly useful given the geographical heterogeneity and sheer scale of Canada.

### Criteria attributes

Criteria attributes provided a repeatable method of comparing pathogens. Although many prioritisation exercises only use numbers for criteria attributes (e.g. [27], [54], [55]), a definition for each attribute was preferred here to minimise the variability in interpretation of criteria between experts. This transparency was particularly important because the tools are multidisciplinary and use a diverse range of criteria about animal and human epidemiology, the influence of climate and the impact on economics and society.

The number of attributes per criteria varied between two and five depending on what was most appropriate for each criterion. Other schemes have used two tiers and up [7], [29], [31], [56], and while more tiers would provide a more differentiated scale, this was balanced against the ability to provide clear definitions for each attribute as well as the availability of information about pathogens with which to reliably select an attribute.

A positive linear scoring method was used to assign values to criteria attributes. Other prioritisation exercises have used similar linear scoring methods, e.g. [8], [20], although non-linear scoring has also been applied when particular answers are considered proportionally more influential than others e.g. [19]. This was not considered necessary in this work, although alternative methods of scoring could be tested if necessary. The number of attributes varied between criterion, therefore sensitivity analysis was performed on the attribute scores in the spreadsheet model. The scores were recalculated so that all attribute values ranged between 0 and 5. The maximum value (worst case) was assigned 5, and all other attributes were assigned standardized values between 0 and 5 depending on the number of attributes for the criteria. There was little change in the pathogen ranking, with the top three, and bottom three pathogens remaining in those positions, for the overall score, for both weighting method 1 and 2.

There were two criteria where a linear scale was not appropriate because the attributes could not be placed into a natural ascending order (pathogen taxonomic group and mode of transmission) and in these cases the experts' modal rank was considered the most appropriate value. An alternative method to account for the apparent bimodal opinion of experts might be to use negative values. This method has been demonstrated in a prioritisation scheme for infectious diseases in Germany, where values of either −1, 0 or 1 are allocated to each criterion (low importance, lack of knowledge or opinion and high importance respectively) [29]. One other important point to note about our scoring system is that some attributes generated a value of zero, in particular when the attribute was ‘not applicable’ (e.g. attribute 1 of criteria B4 and B5) or when the attribute was not a perceived risk (e.g. attribute 1 of criteria A4 and A5). The first three attributes for criteria C3 to C12 were assigned 0 because all of them (lack of information (attribute 1), inhibition of pathogen emergence (attribute 2) or no influence on pathogen emergence (attribute 3)) were deemed low risk for this study. This meant that the score of a pathogen would only increase if it is influenced by climate, while at the same time documenting that an attribute had been assessed even if it generated a score of 0. Finally, ‘unknown’ attributes also generated a value of 0 (e.g. attribute 1 of criteria A6), however if a decision maker preferred to highlight pathogens about which little is known, then a value of 0 could be replaced with a higher value.

### Reliability and improvement

The prioritisation tools proved to be a standardised method to collate information about a pathogen or disease and previous work has shown that MCDA can allow a more complete understanding of the consequences associated with choices [15]. The tools will of course be limited by the reliability and availability of information about pathogens. It is therefore important not only to consider the final outcome, but to consider the process embodied within the tools. A benchmark against which to compare the model results is not possible since there is no absolute measure of potential disease emergence. However, results have been compared to current literature and have been presented to stakeholders and interested parties for discussion and feedback. Suggestions from stakeholders and from the expert group were incorporated during tool design.

One suggestion from experts and from literature [57] was to prioritise likelihood of emergence and impact in two separate assessments. However our prioritisation assessed both because similar studies have demonstrated the importance of assessing impact at the same time as risk [7], [20], [31], because discussion with the authors of these publications stressed the need for them to be included, because economic and social impact have a considerable influence on policy making [58] and because the impact criteria were amongst the most highly weighted by the experts. If a user required a prioritisation based on only a sub-set of the criteria, then they could adopt the relevant criteria for the process.

### Sensitivity analysis

The ‘reduced’ spreadsheet and MACBETH tools included only 10 criteria that focused on current climatic conditions, mode of transmission, severity and fatality in the human population and excluded many criteria related to climate and treatment or control. The ranking of diseases in the spreadsheet tool did not vary noticeably from the full model, suggesting that these criteria played an influential role in the full model.

In comparison, the pathogen ranking did change in the MACBETH tool. We attribute the differences to the method of weighting and scoring. In the ‘reduced’ MACBETH tool it was necessary to standardise the weights of the ten criteria so that they summed to 100. As a result there was more differentiation between criteria weights than in the full model. Cholera increased in rank from eighth to second because the tool focused on transmission and impact on the human population – the highest risk criteria for this disease. These criteria were assigned a relatively high weight in the ‘reduced’ model. Giardiasis and Hantavirus dropped in rank. In the tool containing all criteria, the ranking of these diseases resulted mostly from their disease epidemiology and inability to monitor, treat and control. These criteria were given a relatively low weight or were excluded altogether in this ‘reduced’ tool.

When only a small number of low ranking criteria were excluded in the ‘intermediate’ spreadsheet and MACBETH tools (the lowest ranking criteria were excluded from each criteria group in turn) there was little change in the overall ranking of pathogens. These results, as well as results from the ‘reduced’ models suggest that it might be possible to build a robust tool with fewer criteria than are currently included. While such a tool would be advantageous for a rapid pathogen prioritisation, the criteria would need to be selected carefully to incorporate characteristics of concern, while acknowledging those that had been excluded.

Further work to assess a broader set of pathogens would be a logical next step. In the UK work is on-going to develop the ‘ENHanCEd Infectious Diseases’ (EID2) database [59], (a database detailing all pathogens that are known to infect humans), which, in the future, might be used to generate the raw data for an extensive prioritisation. As well as pathogen ranking, the resulting pathogen scores could be translated into practical recommendations; e.g. a low score indicating pathogens of minimal concern, with higher scores above a certain threshold indicating evaluation is needed, more data is required or a risk assessment is recommended. This approach has been employed in other risk evaluations [31], [57].

### Comparison of Excel and M-MACBETH platforms to develop an MCDA tool

M-MACBETH was selected for its ability to establish quantitative measurement scales based on qualitative judgement. However, we found that we were able to assign our own quantitative values for criteria weights and criteria attributes based on literature and expert input. It was therefore not necessary to use these capabilities to their full extent and we do not include further details about matrix building; details of the mathematical foundations of MACBETH can be found elsewhere [15], [60].

One advantage of M-MACBETH is that it offers a variety of visually attractive ways to compare pathogens (e.g. XY maps and difference profiles). The M-MACBETH program, however, incurs a cost and may require user training. Excel, in comparison, is a widely-used program, and although the criteria, weighting and scoring are predefined, they may be altered easily as necessary. Modifications could be made for example, to focus on particular types of pathogens or could be applied to scenarios in other regions of the world. Further development of the Excel tool through the use of custom code could incorporate some of the features of M-MACBETH, for example, the ability to graphically compare the ranking of multiple pathogens.

## Conclusion

The tools developed here provided a user friendly approach to aid pathogen prioritisation. In particular they were useful for synthesising information about a large number of criteria, they helped provide structure for prioritisation exercises, and they acted as a record of decision making. They can be used to provide a rapid and simple assessment of pathogens by a user who does not require expert knowledge of each pathogen and they can be used to highlight gaps in knowledge. The tools are a novel method of prioritising infectious pathogens according to their probability of emergence in response to climate change. They can incorporate both expert opinion and empirical data into a pathogen ranking system and can be used to identify pathogens that should be investigated more fully.
